# Nickel ion release from dental alloys in two different mouthwashes

**DOI:** 10.15171/joddd.2019.003

**Published:** 2019-04-24

**Authors:** Karim Jafari, Saeed Rahimzadeh, Somayeh Hekmatfar

**Affiliations:** ^1^Department of Prosthodontics, Faculty of Dentistry, Ardabil University of Medical Sciences, Ardabil, Iran; ^2^Dental Research Committee, Faculty of Dentistry, Ardabil University of Medical Sciences, Ardabil, Iran; ^3^Department of Pedodontics, Faculty of Dentistry, Ardabil University of Medical Sciences, Ardabil, Iran

**Keywords:** Corrosion, dental alloys, nickel

## Abstract

***Background***. Mouthwashes are widely used as adjuncts to mechanical oral hygiene procedures. Nonetheless, there is little information regarding the effect of various mouthwashes on the amount of ions released from the nickel-chromium (Ni‒Cr) alloys used in the fabrication of fixed prostheses. Therefore, the present study was conducted to evaluate the effect of two types of mouthwash on the release of Ni ions from dental alloys.

***Methods***. Forty-two disk-shaped specimens were prepared with a diameter of 10 mm and a height of 2 mm. Two mouthwashes were examined in this study: Oral B and Listerine. A control group was also considered using distilled water. Each Ni‒Cr disk was immersed in the mouthwashes and distilled water in polypropylene test tubes, and then incubated at 37°C to simulate the oral temperature. After 45 days of incubation, the samples were tested for Ni ions using inductively coupled plasma mass spectroscopy. Data were analyzed using ANOVA.

***Results***. In the Halita group subjects exhibited 2.04±0.65 reduction in OLS. OLS reduction in the chlorhexidine group was 1.95±0.74. Statistical analysis showed no significant difference between the two groups (P>0.05).

***Conclusion***. As the results indicated, the amount of ion release was within the safe limits in the two experimental groups. However, it is recommended that prescribe Listerine mouthwash should not be prescribed for the patients with a history of Ni allergy.

## Introduction

Metal‒ceramic crown is one of the most widely used fixed restorations in many dental procedures. This restoration offers a predictable esthetic outcome, is associated with sound physical properties and remains in the oral cavity for a long time.^1^ The alloys used for fixed prosthodontics are mostly composed of nickel‒chromium (Ni‒Cr).^2^ The constant contact of these restorations with mucosa, saliva, periodontal tissues and bone highlights the importance of an in-depth analysis of their chemical and physical characteristics and biocompatibility assays for ensuring patient safety.

Biocompatibility is related to corrosion in a biological environment. The release of metal ions during corrosion might lead to several possible consequences, including serious damage to patient's health, allergies, oral lesions and a salty or metallic taste.^3^ Nickel is found in very low concentrations in the human body. An increase in the concentration of this element is hazardous.

According to statistics, around 1 in 10 people is allergic to Ni. Nickel exposure is associated with a number of systemic disorders.^4^ In case of metal contact allergy, exposure to dental crowns could play a role.^5^ Allergens can leach from an alloy and dissolve in the oral environment.^2^ The corrosion of Ni‒Cr alloys occurs by the preferential dissolution of Ni-rich grains.^4^

Temperature and pH affect the corrosion resistance; however, Ni‒Cr alloy is more susceptible to acid attack and results in decreased cell viability, increased oxidative and cellular toxicity levels, and enhanced cytokine inflammatory expression.^6,7^ According to several studies, the corrosion effect is increased by low pH conditions, leading to an increase in leaching of Ni ions into the simulated oral environment.^8-10^

Mercieca studied the corrosion resistance of cast cobalt‒ and Ni‒Cr dental alloys in acidic saliva and concluded that Ni‒Cr alloys are unstable in solution and leach Ni ions.^4^ The use of mouthwashes containing fluoride, chloride or essential oils is occasionally recommended by prosthodontist as an adjunctive therapy to reduce the risk of caries and plaque formation and overcome periodontal conditions, such as gingivitis.^11^ However, only a limited number of studies have investigated the relationship between mouthwashes and metal corrosion of Ni‒Cr alloys.

With this background in mind, the present study was conducted to examine the effects of two types of mouthwashes on Ni-Cr alloy disks by measuring the amount of Ni ions released from these disks when immersed in various types of mouthwash. Knowledge on the amount of ions released could assist the practitioner in prescribing an appropriate mouthwash for the patient’s benefit.

## Methods

**Figure 1:**
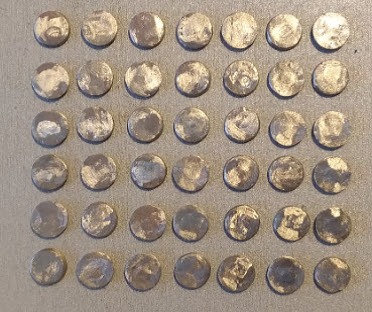
Ni-Cr disc (Suprem Cast V Talladium Inc., Valencia, CA, USA).

The base metal alloy used for this study was Ni‒Cr (Suprem Cast V Talladium Inc., Valencia, CA, USA). For the purpose of the study, 42 disk-shaped specimens were prepared with a diameter 10 mm and a height of 2 mm (Figure 1). All the specimens were polished with silicon carbide paper (400, 800 and 1200 grit). In the next stage, the specimens were cleaned in ethanol and deionized water in an ultrasonic cleaner for 5 minutes to remove the contaminants, and then autoclave-sterilized as previously reported by Xin et al^12^ and McGinley et al.^8^

**Figure 2:**
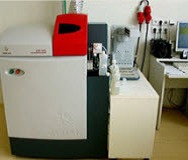
Mass spectroscopy device.

Two mouthwashes were used in this study: ORAL B (Procter and Gamble, Weybridge, London, UK) and Listerine (Johnson and Johnson Healthcare Products, USA). In addition, distilled water was considered as the control group. The compositions of the materials used in our study are presented in Table 1. Each Ni-Cr disk (14 disks in each group) was immersed in the solutions in polypropylene test tubes and then incubated at 37°C to simulate the oral temperature. After 45 days of incubation, the samples were tested for Ni ions using inductively coupled plasma (ICP) mass spectroscopy (Figure 2).

**Table 1:** Materials’ brand name, composition and manufacturer

**Material**	**Compositions**	**Manufacturers**
**Suprem Cast V**	Ni:74% , Cr:14% , Mo:8.5% , Be:1.8% , Al:1.7%	Talladium Inc., Valencia, CA, USA
**Oral B- sensitive**	Sodium fluoride, water, methylparaben, polysorbate 80, sodium saccharin, methyl salicylate, propylparaben, sodium hydroxide, spearmint oil, Menthol	Oral-B Laboratories, London, UK
**Listerine -Cool Mint**	Eucaptol, methyl salicylate, mentho, thymol water, alcohol21.6%	Johnson and Johnson healthcare products, USA

### Statistical Analysis

Kolmogorov-Smirnov test was used to assess the normality of data. Furthermore, one-way ANOVA was utilized to assess the significance of the observed differences in Ni mean values between the two mouthwashes.

## Results

Table 2 presents the means and standard deviations of the released ions. The results of the Kolmogorov-Smirnov test showed normal distribution of data in all the groups (P>0.05). According to ANOVA, there was a significant difference among the three groups (P<0.05). Furthermore, the results of post hoc tests revealed a statistically significant difference between the experimental groups in this regard (P<0.05). The Listerine group exhibited the highest release of Ni ions, compared to the other groups. On the other hand, the control group had the lowest mean value of Ni ion release.

**Table 2:** Mean ± SD of nickel ion release

**Solution (I)**	**Mean ± SD**	**Solution (J)**	**Mean difference (I-J)**	**P-value**
**Oral B**	12.04±1.28	Listerine	-7.62	0.001
		Distilled water	11.88	
**Listerine**	19.67±2.02	Oral B	7.62	0.001
		Distilled water	19.51	
**Distilled water**	0.16±0.012	Oral B	11.88‏-	0.001
		Listerine	19.51‏-	

## Discussion

The knowledge on the degree of metal ion release is important to prevent the associated adverse effects of mouthwashes on the patient, such as toxicity, metallic taste, mucositis, gingival hyperplasia and gingivitis.^13^ Corrosion is an electrochemical reaction on the metal surface, which leads to the release of ions by the metal. This process can occur as a result of internal and external factors. The internal factors affecting corrosion include metal composition and structure. On the other hand, the external factors that could affect corrosion are the biological environment, pH and temperature.^13^

Several studies have investigated the effects of the environmental conditions on the corrosion of alloys used in dental prostheses.^14^ The alloys intended for intraoral use should be resistant to corrosion and chemical degradation.^15^ The alloys utilized in the oral cavity have been reported to leach heavy metals in the saliva under normal conditions. In an in vitro study, metal ion leaching was indicated to be pH-dependent. In another study, Ni and Cr were reported to leach out of metal alloys; however, molybdenum and carbon were more stable.^16^

The use of mouthwashes has recently become popular as an effective method for the prevention and control of caries and periodontal diseases. In addition, mouthwashes are widely used to decrease oral malodor and maintain implants.^17,18^ However, little information is available regarding the effect of different mouthwashes on ion release from the dental alloys. Mouthwashes have been reported to affect the solubility of some restorative materials.^19^

In this study, we examined Ni‒Cr metal disks that are widely used for the fabrication of dental prostheses. The specimens were immersed in two types of mouthwash and distilled water for 45 days and then tested by means of an ICP mass spectrometer. Unlike other methods, such as atomic emission spectrometry, ICP has the advantage of extracting each ion simultaneously and detecting the metals without the interference of other ions.^20^

Mouthwashes are usually used twice a week for about 1 minute. The patients are recommended not to eat, drink or rinse after using a mouthwash. Therefore, the components of mouthwashes can be in contact with the Ni‒Cr crowns for a long time; nonetheless, it is difficult to determine the exact duration of this contact. In the current study, we assumed that the mouthwash was present for 6 hours in a patient’s mouth each time. Consequently, the metal disks were immersed in mouthwashes and incubated for 45 days.^20^

In line with the results of other studies, in the present study, Ni ion release was higher with the use of Listerine mouthwash than with the Oral B mouthwash. Listerine is an alcohol-based mouthwash. In this regard, Erdogana^21^ studied the metal ion release from silver soldering and laser welding caused by different types of mouthwash and reported that NaF+alcohol (Listerine) mouthwashes exhibited the highest amount of metal ion release.

In a study by Kuhta et al,^22^ pH factor was reported to significantly affect ion release. In the study above, more visible ions were released at a pH of 3.5, compared to those at a pH of 6.75 after a 28-day immersion period.Mihardjanti et al^23^ studied Ni and Cr ion release from stainless steel brackets immersed in various types of mouthwash. They reported that Listerine mouthwash resulted in the release of the highest amounts of Ni and Cr ions.

In another study by Mandsaurwala et al,^24^ the greatest amount of metal ion release was observed in the mouthwashescontaining Na and alcohol.Listerine and Oral B mouthwashes have a pH of 4.33 and 5.1, respectively. A lower pH can affect the ion release of appliances and alloys in the oral cavity. Metal is released into the oral cavity with saliva as the medium. This could be influenced by a high chloride mixture in the saliva or the intake of various foods and drinks with low pH levels. Moreover, the characteristics of the saliva might change based on the patient’s health and time of day.^25^

In the current study, the mouthwashes were examined in a static condition. However, more metal release could occur in the real life due to the fluidity of saliva in the mouth and removal of oxide layers by tooth brushing. Kerosuo et al^26^ reported a great amount of release after using an oral functioning simulator apparatus to simulate the dynamic conditions of the mouth.In our study, the level of ion released from Ni‒Cr disks was within the safe limits recommended by the World Health Organization. The recommended maximum limit for Niis 200‒300 μg.^24^

## Conclusion

The Ni‒Cr alloy is susceptible to corrosion when exposed to mouthwashes. According to the findings, the amount of ion release was within the safe limit. Based on our results, further randomized clinical trials are recommended to evaluate the exact effect of mouthwashes on ion release. Therefore, Listerine mouthwash should be used cautiously in patients allergic to Ni.

## Acknowledgments

This study was part of a doctoral thesis (#84). The authors would like to express their gratitude to the Research Deputy of Ardabil University of Medical Sciences, Ardabil, Iran, for financially supporting this study.

## Conflict of Interests

The authors declare no conflict(s) of interest related to the publication of this work.

## Authors’ contributions

KJ was responsible for conceiving, designing and interpretation of data. SR was responsible for acquisition of data and editing. SH drafted the manuscript and collected the data.

## Funding

The study was funded by Ardabil University of Medical Sciences.

## Ethics approval

Not applicable.
